# Patients undergoing elective and oncofertility preservation respond similarly to controlled ovarian stimulation for fertility preservation

**DOI:** 10.5935/1518-0557.20210080

**Published:** 2022

**Authors:** Ariane Tieko Frare Kira, Marta Ribeiro Hentschke, Natália Fontoura de Vasconcelos, Talita Colombo, Vanessa Devens Trindade, Alvaro Petracco, Bartira Ercília Pinheiro da Costa, Mariangela Badalotti

**Affiliations:** 1 Fertilitat - Reproductive Medicine Center, Porto Alegre, Brazil; 2 School of Medicine, Pontifical Catholic University of Rio Grande do Sul, PUCRS

**Keywords:** cryopreservation, fertility preservation, oncofertility, ovarian stimulation, neoplasms

## Abstract

**Objective:**

Outcome data for oocyte vitrification for fertility preservation are still scarce despite the scientific and technological advances. Studies suggest that patients with cancer have worse outcomes regarding mature vitrified oocytes when compared to healthy patients. Thus, the objective of this study was to evaluate and compare the oocyte vitrification response in patients undergoing elective and oncofertility preservation.

**Methods:**

The ovarian stimulation response was verified by a cross-sectional and observational study, analyzing data from 367 patients between 2009 and 2018, which were divided into elective group (EG; n=327) and oncofertility group (OFG; n=40). The number of follicles, oocytes, mature oocytes, and duration of the cycle was evaluated, which were compared with clinical and ovarian stimulus data between groups.

**Results:**

A significant difference in women's age (31.3±5.8 vs. 37.0±2.9 years; *p*<0.01) and basal values of Follicle Stimulating Hormone (FSH), (4.0 [3.3 - 6.2] vs. 9.0 [5.4 - 9.9] mIU/mL; *p*<0.01) were observed. When adjusting data for age, FSH and Gonadotropin-releasing Hormone protocols, no significant difference in the number of vitrified mature oocytes between the two groups were observed (6.0 [3.0-11.0] vs. 7.0 [3.0-12.0]; *p*=0.11).

**Conclusions:**

Thus, patients undergoing elective and oncofertility preservation seem to respond similarly to controlled ovarian stimulation for fertility preservation. Breast cancer was the most frequent in the OFG (67%).

## INTRODUCTION

### Fertility preservation

There have been many advances in the strategies for fertility preservation to ensure higher chances of conceiving. However, despite all scientific and technological advances, age persists as the factor with the most significant impact on the prognosis of female fertility. It is well established that the quantity and quality of oocytes decrease throughout a woman's lifespan, especially from the age of 35, being expressed through the progressive reduction in the ovarian reserve (Stoop et al., 2014). Age is also related to lower chances of conceiving naturally, higher rates of spontaneous abortions, more embryonic chromosomal abnormalities, and lower success rate in assisted reproduction treatment (González-Foruria et al., 2016; Frederiksen et al., 2018).

### Ovarian reserve

The ovarian reserve can also be seriously threatened by surgical, radiotherapy or chemotherapy procedures, which, due to the action of cytotoxic agents and hormonal suppressants, lead to temporary or permanent infertility (Loren et al., 2013; Muñoz et al., 2016). Thus, maternity delay and the increased incidence of malignant diseases are the most jeopardizing conditions for reproductive potential in women (Cobo et al., 2018).

### Elective and oncofertility oocyte preservation

Despite the growing number of publications, studies are conflicting about oocyte freezing results in patients with and without cancer. Some studies suggest worse outcomes in patients with cancer regarding the number of mature vitrified oocytes when compared to healthy patients (Friedler et al., 2012; Domingo et al., 2012; Pal et al., 1998) while others show a similar response to the ovarian stimulation for fertility preservation in both groups, once the number of vitrified oocytes was similar (Moraes *et al*., 2019). Thus, the objective of this study was to evaluate and compare the oocyte vitrification response in patients undergoing elective and oncofertility preservation, in a southern Brazilian population. The results will not only contribute to the improvement of the technique but also help in the optimization of counseling for this population.

## MATERIAL AND METHODS

### Study Design

Observational, cross-sectional, and historical study.

### Patient sample, study period and location

Patient sample, study period and location Patients who underwent oocyte vitrification between 2009 and 2018 at a Reproductive Medicine Center in Porto Alegre, Brazil. Patients with complete medical records were included and divided into an elective group (EG; n=327) and a oncofertility group (OFG; n=40), totalizing 367 patients.

The study was approved by the Research Ethics Committee of the Pontifical Catholic University of Rio Grande do Sul, PUCRS (Protocol 2.828.376).

### Study variables

The main clinical and demographic characteristics, ovarian stimulation characteristics, number of follicles, mature oocytes, and duration of treatment were compared between groups.

### Controlled-ovarian stimulation

For the controlled-ovarian stimulation, recombinant Follicle-Stimulating Hormone (FSH-r), Human Menopausal Gonadotropin or Clomiphene Citrate were prescribed.Letrozole was added for some breast cancer patients to avoid the hyperestrogenism state caused by the multi follicular growth. The final oocyte maturation was triggered with GnRH agonist or Human Chorionic Gonadotropin (hCG). The oocyte retrieval was performed after 35h of hCG triggering. Both groups underwent treatment with agonist or antagonist Gonadotropin-releasing Hormone (GnRH) protocol, according to medical indication.

### Statistical Analysis

The analysis was performed using the SPSS version 21.0 program. Quantitative variables were described as mean and standard deviation (SD) or median and interquartile range (IQR), while categorical variables were expressed as absolute and relative frequencies. The Chi-square test was used to compare proportions. Comparisons of means between groups were performed using Student's t-test, or Mann-Whitney test in case of asymmetry. Generalized linear models were used to control confounding factors. Data were adjusted by women's age, FSH, and GnRH protocol.The null hypothesis was rejected when *p*<0.05.

## RESULTS

### Clinical and laboratory characteristics

The main clinical and laboratory characteristics are described in Table 1. The women's age in OFG was significantly lower compared to EC (31.3±5.8 vs. 37.0±2.9 years; *p*<0.01) as well as the baseline FSH measurement (4.0 [3.3 - 6.2] vs. 9.0 (5.4 - 9.9) mIU/mL; *p*<0.01). The presence of a partner was significantly higher in OFG (25 [62.5%] vs. 65 [19.9%]; *p*<0.001).

**Table 1.** Baseline characteristics of the patients subdivided into an Elective Group and Oncofertility Group.

Variable	Total (n=367)	Elective Group (n=327)	Oncofertility Group (n=40)	*p*
**Age (years), mean ±SD**	36.4±3.8	37.0±2.9	31.3±5.8	<0.001†
**Age range (years), n (%)** ≤35 years >35 years	113 (30.8) 254 (69.2)	83 (25.4) 244 (74.6)	30 (75) 10 (25)	<0.001§
**Partner, n (%)**	90 (24.5)	65 (19.9)	25 (62.5)	<0.001§
**FSH (mUI/mL), median (IQR)**	6.8 (5.4–9.7)	6.9 (5.4–9.9)	4 (3.3–6.2)	<0.001‡
**AMH (ng/mL), median (IQR)**	1.5 (0.5–2.8)	1.5 (0.5–2.7)	2.7 (0.7–9.8)	0.313‡
**BMI (kg/m^2^), mean ±SD**	22.3±3.7	22.2±3.7	23.3±3.2	0.324†

FSH: Follicle-stimulating hormone; AMH: Anti-Mullerian hormone; BMI: Body mass index. IQR: Interquartile range. The *p*-values correspond to the tests:

† Student’s t-test,

‡ Mann-Whitney or

§ Chi-square.

There was no statistically significant difference between the groups regarding the GnRH protocol, choice of gonadotropin, trigger, or stimulus duration. The GnRH antagonist protocol was the most used in both groups (80.1%), and FSH-r was used in 80.4% of cycles, as described in Table 2. Letrozole was added for 20 breast cancer patients (74%).

**Table 2.** Characteristics of controlled-ovarian stimulation in the Elective Group and Oncofertility Group.

Variables	Total (n=367)	Elective Group (n=327)	Oncofertility Group (n=40)	*p*
**Gonadotropin–n (%)** FSH-r hMG CC	295 (80.4) 69 (18.8) 3 (0.8)	267 (81.7) 57 (17.4) 3 (0.9)	28 (70) 12 (30) 0 (0)	0.138§
**GnRH Protocol–n (%)** Agonist Antagonist	73 (19.9) 294 (80.1)	61 (18.7) 266 (81.3)	12 (30) 28 (70)	0.137§
***Trigger*–n (%)** hCG GnRH agonist	311 (84.7) 56 (15.3)	274 (83.8) 53 (16.2)	37 (92.5) 3 (7.5)	0.225§
**Stimulus duration (days)**	14.0±1.9	14.0±1.9	14.2±2.1	0.863†

FSHr: Recombinant Follicle-stimulating hormone; hMG: Human menopausal gonadotropin; CC: Clomiphene citrate; GnRH: Gonadotropin-releasing hormone; hCG: Human chorionic gonadotropin. The *p*-values correspond to the tests:

† Student’s t-test and

§ Chi-square test.

There was not statistically difference between the two groups when comparing the number of aspirated follicles, the number of oocytes obtained, the number of mature oocytes even after adjusting for confounding factors using a generalized linear model for age, baseline FSH and GnRH protocol.

When evaluating the percentage of matured oocytes (MII) obtained in the groups (total number of MII/total number of oocytes), there was a higher proportion of MII for the EC with significance after adjustment for age, FSH and GnRH protocol. However, when considering the results according to the age group, there was no longer a significant difference between groups.

### Ovarian follicular aspiration

The main results of ovarian follicular aspiration in the EC and OFG are described in Table 3.

**Table 3.** Results of ovarian follicular aspiration in the Elective Group and Oncofertility Group.

Variable	Total (n=367)	Elective Group (n=327)	Oncofertility Group (n=40)	*p*	*P*adjusted*
Retrieved Follicles - median (IQR)	12 (8–18)	12 (8–18)	15 (7–20)	0.289	0.052‡
Retrieved oocytes - median (IQR)	8 (5–13)	8 (5–13)	9 (5–17)	0.347	0.171‡
**Number of matured oocytes** **(MII) - median (IQR)** Total ≤ 35 years > 35 years	6 (3–11) 8 (4–13)(n=113) 5 (3–9)(n=254)	6 (3–11) 7 (4–13)(n=83) 5 (3–9)(n=244)	7 (3–12) 8 (6–13)(n=30) 2.5 (2–8)(n=10)	0.481 0.669 0.079	0.113‡ 0.085‡ 0.231‡
**MII ≥ 8 - n (%)** ≤ 35 years > 35 years	148 (40.3) 57 (50.4) 91 (35.8)	130 (39.8) 41 (49.4) 89 (36.5)	18 (45.0) 16 (53.3) 2 (20.0)	0.640 0.876 0.502	0.701§ 0.448§ 0.914§
**Percentage of MII oocytes** **(MII/Retrieved oocytes)** **mean ±SD**	71.6±23.9	72.1±23.7	67.4±25.9	0.242	0.028†
**Percentage of MII oocytes** **(MII/Retrieved oocytes)** **mean ±SD** ≤ 35 years > 35 years	72.8±22.6 71.0±24.6	72.2±23.2 71.4±24.2	69.2±23.7 62.0±32.3	0.301 0.240	0.192†

MII: Mature oocytes in metaphase II;

* Adjusted by age, FSH and GnRH protocol through a generalized linear model.

† Student’s t-test,

‡ Mann-Whitney or

§ Chi-square.

### Types of cancer in Oncofertility group

Breast cancer was the most frequent (67%), followed by cervical cancer (12%) and ovarian cancer (7%). The other subtypes were 14% and included lymphoma, colon, melanoma, neuroendocrine tumor, and astrocytoma. The age of patients with breast tumors proved to be significantly higher when compared to the age of patients with other cancer subtypes (33.7 vs. 26.2 years, p<0.01). However, no statistically significant differences in the number of mature eggs were observed between the groups (Table 4).

**Table 4.** Mature oocytes vitrified results according to age and cancer subtype.

Subtype	Breast	Others	*p*
Cases - n (%)	27 (67%)	13 (33%)	
Age (years) - mean ±SD	33.7±4.1	26.2±5.5	<0.001†
MII - median (IQR)	7 (2–12)	8 (5.5–12.5)	0.407‡

MII: Mature oocytes in metaphase II; The *p*-values correspond to the tests:

† Student’s t-test and

‡ Mann-Whitney.

## DISCUSSION

The presented study aimed to evaluate and compare the oocyte vitrification response in patients undergoing elective and oncofertility preservation, in a southern Brazilian population. As the main result it was observed that both groups of patients responded similarly to controlled ovarian stimulation for fertility preservation.

### Fertility preservation and age-related fertility

The concern with age-related fertility decline and the diagnosis of cancer are the primary motivation for fertility preservation, which explains the difference observed in women's age (Cobo et al., 2018; Friedler et al., 2012; Moraes et al., 2019). The extensive number of cycles performed for EG in contrast to OFG reflects the trending demand for fertility preservation related to social reasons. It also leads to a reflection on the discreet number of patients who are still not referred for reproductive counseling after a cancer diagnosis. The proportion of cancer patients in this study was 10.9%.

### Clinical and laboratory variables

#### Women's age

The justification for the age difference leads to questions about the motivation for freezing. The presence of a partner was significantly lower for the EC. The profile outlined by this study points out that most cancer patients had never been pregnant or even thought about the intended family design before diagnosis, and the presence or absence of a partner did not seem to interfere with the decision. Nevertheless, the EG motivation is mainly due to aging, the absence of a partner, and the desire to not interrupt professional development or academic titles (Moraes et al., 2019; Kim et al., 2018; Hammarberg et al., 2017; Baldwin et al., 2019). Rarely these patients look for fertility preservation before the age of 35 years, after that, there is a known decrease in the ovarian reserve and pregnancy results inherent to advanced age. In contrast, a cancer patient, after being diagnosed, is promptly referred for reproductive counseling with little or no delay in starting the cryopreservation process.

#### Baseline FSH and AMH levels

The serum FSH was significantly lower in the OFG, while AMH was higher in this group, with no statistical significance. This information corroborates the findings in the literature and has a direct correspondence with the age of the patients, which was lower in the OFG (Cobo et al., 2018; Moraes et al., 2019; Cardozo et al., 2015).

#### GnRH protocol

In agreement with other studies, there was no significant difference between groups regarding the GnRH protocol, choice of gonadotropin, trigger, or stimulus duration (Cobo et al., 2018; Friedler et al., 2012; Cardozo et al., 2015).

#### Controlled-ovarian stimulation

When the number of aspirated follicles was compared, the number of oocytes obtained and the number of mature oocytes in MII, even after being adjusted by women's age, baseline FSH and GnRH protocol, showed no significant difference between groups. Similar findings have been observed in previous studies (Cobo et al., 2018; Moraes et al., 2019; Cardozo et al., 2015; Quinn et al., 2017). However, when considering the percentage of mature oocytes over the number of total oocytes retrieved, a higher proportion of vitrified mature oocytes was noted in the EC after adjustment. The significance was lost after the 35 years old cut off was set. Some considerations must be noted to explain these results. The number of women in the OFG is much lower than the EC and can be considered a limitation, although the study included all patients attending the clinic. These data can raise questions about whether the number of cancer patients is insufficient to support these findings strongly.

#### Demand for cancer cryopreservation

The demand for cancer cryopreservation is still notably more discreet when compared to elective freezing, even when considering the progressively higher incidence of malignancies in women of reproductive age. Although the effectiveness of the technique has already been well demonstrated, the number of patients who effectively preserve their reproductive potential could be more significant.

In a recent publication by a European group, the proportion of patients who have frozen eggs due to oncological reasons has remained stable in recent years, corresponding to only 2% of the total number of procedures (Cobo et al., 2018). In contrast, the number of elective procedures has seen a dramatic increase year after year. This fact has been witnessed by several other countries and even in the reproductive medicine center where this research was carried out.

The main justifications for this phenomenon would be, first, because the number of cancer diagnoses is much lower than the number of healthy women who delay motherhood. Second is that the oncologist and the gynecologist have a central role in this phenomenon as many professionals have little or no knowledge of the technique. They either fear the delay in starting cancer treatment or the worsening of tumor staging, consider the procedure financially unfeasible, or do not even think about the possibility. Healthcare professionals must also know about the technique safety, the non-delay in starting antineoplastic treatment, and that the number of mature oocytes obtained in cancer patients seems to have no difference when compared to women without cancer (Cobo et al., 2018; Cardozo et al., 2015; Akel et al., 2020). There is an urgent need to demystify the issue so that more women can benefit from consistent information and preserve their oocyte heritage.

#### Types of tumors

The OFG was composed of different types of tumors in different locations and stages. It was not possible to individually analyze the different cancer subtypes, gene mutations, or staging, basically due to the small number of patients who froze until the end of the study and owing to the urgency to start antineoplastic treatment. Immunohistochemical, molecular analysis, or the complete characterization of staging were not fully concluded until the patient's arrival for reproductive counseling. Thus, it can be questioned whether any patient with a more aggressive tumor might have had a negative impact on the results.

## CONCLUSION

The results presented by this study showed that healthy patients and patients with cancer seem to respond similarly to ovarian stimulation for fertility preservation. These findings support the maintenance of the cryopreservation recommendation for these patients. The patients who seek this alternative treatment, wish to preserve, above all, their autonomy and their future perspectives, and this intention is invaluable. The importance of exploring cryopreservation, especially in oncofertility is to provide answers that can improve the technique to its maximum capacity. Thus, these findings contribute to enable well-founded counseling and help ensure patients the best possible treatment within the limitations of the technique.
